# Two Octaves Supercontinuum Generation in Lead-Bismuth Glass Based Photonic Crystal Fiber

**DOI:** 10.3390/ma7064658

**Published:** 2014-06-19

**Authors:** Ryszard Buczynski, Henry Bookey, Mariusz Klimczak, Dariusz Pysz, Ryszard Stepien, Tadeusz Martynkien, John E. McCarthy, Andrew J. Waddie, Ajoy K. Kar, Mohammad R. Taghizadeh

**Affiliations:** 1 Institute of Electronic Materials Technology, Wólczyńska 133, 01-919 Warsaw, Poland; E-Mails: mariusz.klimczak@itme.edu.pl (M.K.); dariusz.pysz@itme.edu.pl (D.P.); ryszard.stepien@itme.edu.pl (R.S.); 2 Institute of Photonics and Quantum Sciences, Heriot-Watt University, Edinburgh EH14 4AS, UK; E-Mails: jem17@hw.ac.uk (J.E.M.); A.Waddie@hw.ac.uk (A.J.W.); A.K.Kar@hw.ac.uk (A.K.K.); M.Taghizadeh@hw.ac.uk (M.R.T.); 3 Fraunhofer Centre for Applied Photonics, 347 Cathedral Street, University Centre, Glasgow G1 2TB, UK; E-Mail: henry.bookey@fraunhofer.co.uk; 4 Institute of Physics, Wrocław University of Technology, Wyb.Wyspiańskiego 27, 50-370 Wrocław, Poland; E-Mail: Tadeusz.Martynkien@pwr.edu.pl

**Keywords:** soft glass, supercontinuum generation, photonic crystal fibers

## Abstract

In this paper we report a two octave spanning supercontinuum generation in a bandwidth of 700–3000 nm in a single-mode photonic crystal fiber made of lead-bismuth-gallate glass. To our knowledge this is the broadest supercontinuum reported in heavy metal oxide glass based fibers. The fiber was fabricated using an in-house synthesized glass with optimized nonlinear, rheological and transmission properties in the range of 500–4800 nm. The photonic cladding consists of 8 rings of air holes. The fiber has a zero dispersion wavelength (ZDW) at 1460 nm. Its dispersion is determined mainly by the first ring of holes in the cladding with a relative hole size of 0.73. Relative hole size of the remaining seven rings is 0.54, which allows single mode performance of the fiber in the infrared range and reduces attenuation of the fundamental mode. The fiber is pumped into anomalous dispersion with 150 fs pulses at 1540 nm. Observed spectrum of 700–3000 nm was generated in 2 cm of fiber with pulse energy below 4 nJ. A flatness of 5 dB was observed in 950–2500 nm range.

## 1. Introduction

A considerable amount of research work related to supercontinuum generation in photonic crystal fibers (PCFs) has, to date, been focused on the more conventional, mainly silica-based fibers operating in the visible and near-infrared (NIR) [1]. Various step-index fibers and PCFs based on soft glasses have also been used successfully for broadband supercontinuum generation [2,3,4,5]. Typically, high nonlinearity of soft-glass PCFs comes at a cost of high attenuation [6]. For this reason, femtosecond pumping regime, where short samples of fibers are used, is preferred [5]. Only ZBLAN glass offers low attenuation in the mid-infrared region, going down to even below 0.1 dB/m in 2–3.5 μm wavelength range [7]. However development of a tellurite fiber with glass attenuation as low as 0.5 dB/m in the range of 600–4500 nm has been recently reported [8]. The use of broadband mid-infrared (mid-IR) sources in sensing and spectroscopy has seen a tremendous increase in the recent years. Silica glasses used for standard fiber fabrication cannot be used for this purpose, due to their limited transmission above 2.5 µm. Several promising mid-IR optical glasses, such as selenides and sulphides, crystallize easily during thermal handling in the fiber drawing process. Toxic compounds are also used as dopants to enhance their nonlinearities [9]. The fibers made of these glasses are extremely fragile, degrade with time as well as darken during exposure to high power laser pulses [10,11]. Therefore, from the point of view of both the fabrication and the subsequent deployment of the fiber in a biosciences application, the use of tellurite and/or heavy metal oxide glasses is an interesting approach [12]. Heavy metal oxide glasses have limited transmission in the infrared—with respect to chalcogenide glasses—extending up to 6 µm [13]. On the other hand, heavy metal oxide glasses are transparent also in the visible part of spectrum. A glass of this type enables development of supercontinuum sources that cover the visible, NIR and, in part, mid-IR bands. Recently, successful realizations of mid-IR supercontinuum generation in PCFs and conventional fibers have been reported by several groups in lead silicate [2], ZBLAN [3] and tellurite [4] glasses. However, in general, the spectra demonstrated in these experiments were rather irregular with reported differences in output power across the spectra of 20 dB over 2700 nm range [3], 40 dB over 2500 nm range [2] and 24 dB over 4000 nm [4]. Practical use of supercontinuum with such large variation of intensity in the spectrum is limited, which prompts further research for enhanced spectral flatness.

In this paper we present supercontinuum generation in a PCF fabricated using an in-house synthesized lead-bismuth-gallate glass labeled as PBG-08, with rheological and transmission properties optimized in a spectral range of 500–4800 nm. According to our best knowledge, this glass shows the highest nonlinear refractive index among heavy metal oxide glasses dedicated for fiber drawing [10]. Previously we reported a broadband supercontinuum generation in a multimode PCF based on the same glass in the range of 700–2500 nm pumped with an optical parametric oscillator generating 120 fs pulses (pulse energy of 40 nJ) at 1540 nm [14]. The newly developed fiber has been redesigned to limit guidance to the fundamental mode only. As a result a broader supercontinuum is reported in the range 700–3000 nm, using 4 times lower energy pulses for pumping.

Recently we have reported successful use of the same fiber for an octave spanning supercontinuum (900–2400 nm), under pumping with Er-doped fiber laser, which was passively mode-locked with a graphene saturable absorber and comprised a chirp-pulse amplification (CPA) block, generating 850 fs pulses at 1560 nm [15] and 400 fs [16].

To our knowledge this is the broadest supercontinuum reported up to now in heavy metal oxide glasses. An over two-octave spanning supercontinuum in the range 700–3000 nm is demonstrated in an improved design of the fiber, at a similar pump wavelength, but using shorter pump pulses. Linear and nonlinear characterization data on the fiber is shown and discussed and a general comparison of supercontinuum pumping performance demonstrated in this work is given in relation to previous experiments. This approach would allow the use of cost-efficient and robust 1560 nm femtosecond fiber lasers as pump sources in the near future.

## 2. Nonlinear Single Mode Photonic Crystal Fiber

**Figure 1:**
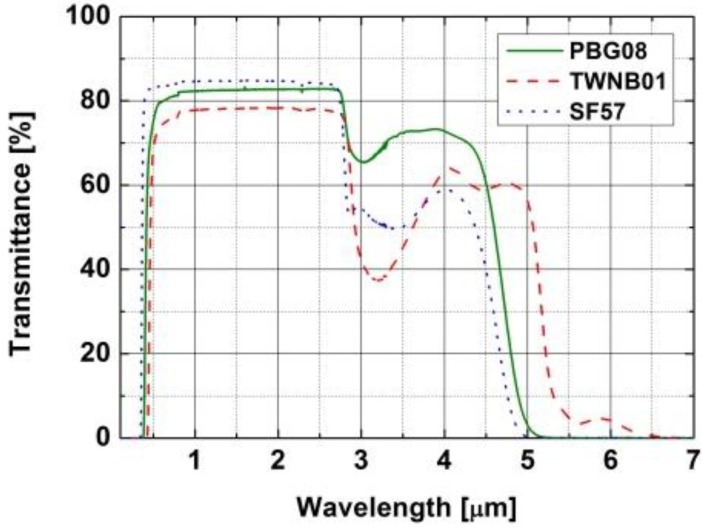
Transmission of lead-bismuth-galate (PBG08) glass. Transmission of tellurite TWNB-01 and commercial lead-silicate SF57 glasses are shown as reference. Bulk sample thickness in all measurements was 2 mm.

The lead-bismuth-gallate glass (PBG-08) selected for PCF development has composition (mol%): 40% SiO_2_, 30% PbO, 10% Bi_2_O_3_, 13% Ga_2_O_3_, 7% CdO and a transmission window extending from 500 nm to about 4800 nm, a high refractive index (more than 2.0) and a high nonlinear refractive index of n_2_ = 4.3 × 10^−19^ m^2^/W, which was measured with the z-scan method at the wavelength of 1240 nm [6]. Figure 1 shows transmission spectra of the PGB-08 glass, with transmission spectra of our tellurite glass and a commercial SF57 glass for reference. Transmission in the 3.0 µm wavelength area is decreased due to presence of OH^−^ ions in the glass, since no purification of glass was applied.

**Figure 2:**
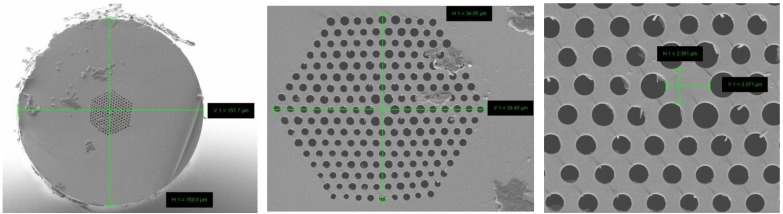
SEM images of single mode photonic crystal fiber made of lead-bismuth-gallate glass. Recorded dimensions of the fiber structure (horizontal × vertical): out fiber dimension 157.7×159.8 µm; photonic cladding 34.85×39.45 µm; fiber core 2.361 × 3.071 µm.

The fabricated PCF consists of eight rings of holes with a lattice constant of Λ = 2.4 µm and relative hole size d/Λ = 0.73 in the first ring and d/Λ = 0.54 in the remaining rings (Figure 2). Some variation between air-hole diameters in the outer rings was observed. Since these variations are small (less than 10% of their diameter), their impact on global fiber properties is negligible. The diameter of the core is 3.1 µm with a photonic cladding diameter of 39.4 µm. The total fiber diameter is 159.8 μm.

**Figure 3:**
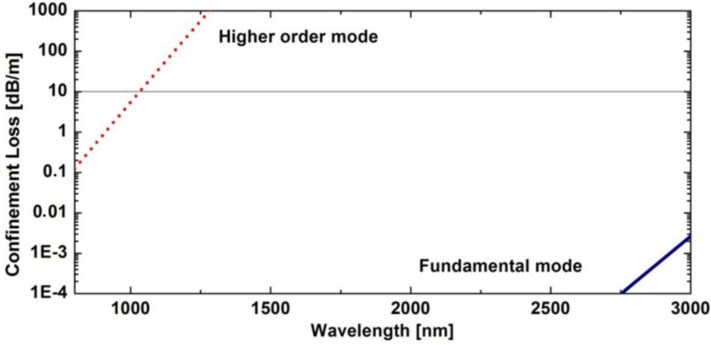
Confinement losses *versus* wavelength, computed using finite element method for the fundamental and higher order mode of the developed photonic crystal fibers (PCF).

The lattice constant and air-hole diameter in first ring determines dispersion properties of the fiber. In the considered case optimum dispersion is obtained for a lattice constant of Λ = 2.4 µm and a relative hole size d/Λ = 0.73. If the remaining rings of air holes would have similar parameters, the fiber would become multimode [17,18]. To avoid this, we reduced the relative hole size to d/Λ = 0.54 in the outer rings. As a result, confinement losses of higher order modes increased significantly by more than 3 orders of magnitude. Confinement losses computed for the fiber using finite element method and SEM image (Figure 2) are compared in Figure 3. At the same time dispersion properties of the fundamental mode are maintained since they are determined by parameters of the first, inner ring.

**Figure 4:**
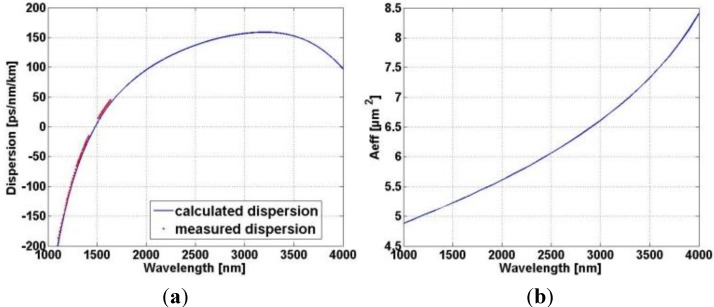
(**a**) Measured dispersion of the fundamental mode (dots) and calculated dispersion for real structure (solid line) of developed nonlinear PCF; (**b**) calculated wavelength dependence of the effective mode area of the fundamental mode.

According to the design, as well as to experimental results, the fiber guide only the degenerated fundamental mode for wavelengths beyond 1100 nm. We measured chromatic dispersion for the fundamental mode of the fabricated fiber, using the white-light spectral interferometric method in a Mach-Zehnder configuration [19]. The measurement results perfectly matches the predicted fiber dispersion calculated with finite element method based on real fiber structure obtained with SEM image (Figure 4a). In addition, an effective mode area is calculated to be used for further numerical modeling of supercontinuum generation.

## 3. Experimental Results

**Figure 5:**
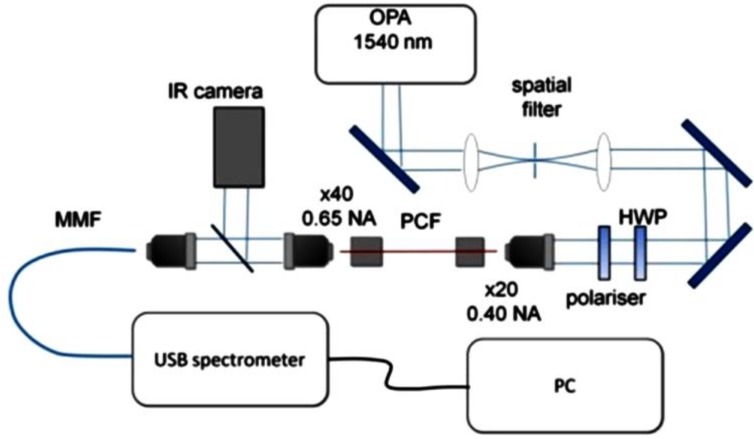
Measurement setup.

For supercontinuum generation we used a Spectra Physics OPA800c optical parametric oscillator (Spectra Physics, Santa Clara, CA, USA) with 1 kHz repetition rate, pumped by a regeneratively amplified seed source from a mode-locked Ti:Sapphire oscillator (Figure 5). The OPA output was tuned to 1540 nm, pulse width was 150 fs. The system was delivering 12 nJ pulses. For fiber coupling, we used a ×20 microscope objective with a numerical aperture of 0.2, which allowed for about 20%–25% coupling efficiencies with the fiber. The near-field mode profile was imaged onto an IR Vidicon camera (Sofradir EC, Fairfield, NJ, USA) for alignment monitoring. Two different USB spectrometers were used. An Ocean Optics NIR512 (Ocean Optics, Dunedin, FL, USA) was used to record the NIR region up to 1700 nm and a BWTek BTC500 Fiber Coupled PbS Array Spectrometer (B&W Tek, Inc., Newark, DE, USA) was used to cover wavelengths between 1000 and 3000 nm. A thermal source was used to calibrate the two spectrometers and the spectral ranges covered meant that no filtering was needed to remove higher grating order radiation. The limit of 3000 nm was set by the spectrometer performance. The output from the OPA was spatially filtered before passing through the half-wave plate-polarizer combination, that are used to vary the incident pulse energy.

**Figure 6:**
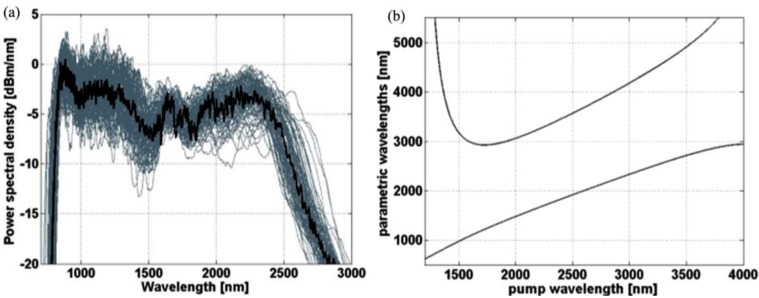
(**a**) Numerically generated supercontinuum spectrum (solid line, average over 100 shots) with numerically generated full range of intensity fluctuations (faded background); (**b**) FWM phase-matching curves calculated for 4 nJ of launched pump energy.

**Figure 7:**
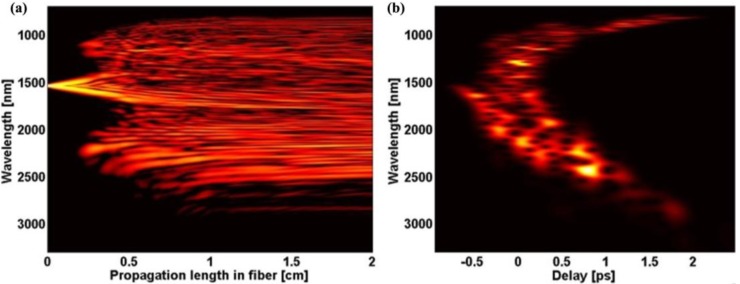
(**a**) Numerically generated evolution of supercontinuum spectrum along the length of PCF; (**b**) corresponding numerical spectrogram at the output of PCF.

Data measured and calculated for the developed PCF in the previous section was used to predict numerically superpcontinuum performance and to estimate fiber length for the experimental work. Numerical model was based on code proposed by Travers *et al.* [20], which we extended to include wavelength-dependent loss and effective mode area of the fiber. We used Raman response parameters of τ_1_ = 5.5 fs and τ_2_ = 32 fs based on a measured Raman gain spectrum in the PBG-08 glass (first order Raman shift at 29 THz), while Raman fractional contribution to nonlinearity in the modeling was set to f_R_ = 0.05. We also included pump noise in the model, taking into account one-photon-per-mode and spectral linewidth of the pump pulse, as proposed by Frosz [21]. One hundred single-shot spectra with random pump noise were calculated in the frequency domain (Figure 6a). The simulations show that spectral broadening should occur at the very first millimeters of PCF and bandwidth deteriorates due to attenuation at fiber lengths of around 5 cm. We decided to use a 2 cm long sample of fiber in the final calculations, and later in the actual experiment. This finds support in combination of relatively short pump pulse and high disposed energy of the pump, and also stems from a desire to avoid fiber attenuation, as well as from some cutting convenience. Numerical spectra generated for 4 nJ (25% of disposed 12 nJ) of input pulse energy are shown in Figure 6 and corresponding evolution of spectrum along the fiber and the output spectrogram are presented in Figure 7.

Numerical supercontinuum bandwidth covers a range of 700–2800 nm in a 20 dB range. At about 5 mm of propagation, energy is transferred to sidebands at around 1000 nm and 2400 nm, which quickly merge with the central area. In order to identify these sidebands, we calculated phase-matching condition for the four-wave mixing (FWM) process, κ = 0, where the phase mismatch κ can be expressed as [22]:


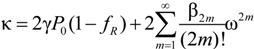
(1)

The resulting phase-matching curves, shown in Figure 6b, indicate a rather broad bandwidth which can be covered by parametric wavelengths generated in a degenerate FWM process in this fiber. For input pulse energy of 4 nJ, it extends from around 1000 nm to about 2800 nm for pump wavelengths close to the used supercontinuum pump wavelength. The two rapidly evolving sidebands of spectrum, indicatory of modulation instability, can be seen in Figure 7a. The Stokes parametric wavelength curve in Figure 6b falls further in the infrared, than the long-wavelength sideband of numerically generated supercontinuum in Figure 6a, which means that pulse energy assumed to calculate phase-mismatch was too high. This discrepancy is assigned to two reasons. The excess energy, which would otherwise constitute a broader FWM bandwidth, in this process is transferred to a dispersive wave across the ZDW at 1500 nm, which can be seen in the short-wavelength, trailing part of the numerical spectrogram in Figure 7b. Parts of it then propagate along the fiber with similar delays as the long-wavelength components, which results in frequency beating appearing as “holes” in the long-wavelength part of spectrogram. The second, non-exclusive explanation for the discrepancy between spectral location of supercontinuum long-wavelength tail and the calculated location of FWM Stokes wavelength, stems simply from fiber attenuation, where some input pulse energy is absorbed by the fiber and cannot participate in the FWM process. Assignment of modulation instability as the primary mechanism in formation of spectrum is supported by the characteristic length scales of the fiber. The nonlinear lengthscale *L*_NL_ = 1/γ/*P*_0_, with γ standing for the nonlinear coefficient and P_0_ for pump peak power, equals 1.3 mm and roughly coincides with the location of onset of the MI sidebands in Figure 7a. The dispersive lengthscale *L*_D_ = *t*_0_^2^/|β_2_| is about 80 cm, which is an order of magnitude higher than the length of the nonlinear fiber. This results in a soliton order *N*_sol_ = (*L*_D_/*L*_NL_)^1/2^ of around 24. Consequently the soliton fission length *L*_D_/*N*_sol_ of 3.3 cm is longer than the length of the fiber.

**Figure 8:**
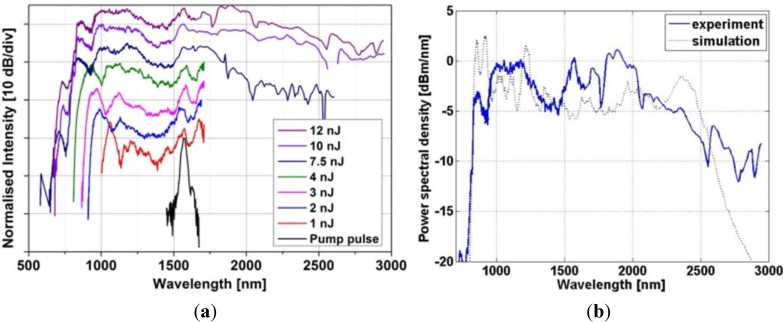
(**a**) Measured supercontinuum spectra in a 2 cm long PCF (pump: 150fs, 1540 nm). Spectra are shown in the order from bottom to top, beginning with the pump pulse; (**b**) measured spectrum (solid trace) and numerical result (dotted trace) for 3.8 nJ pump pulse energy (in-coupled).

Supercontinuum spectra experimentally recorded in the developed fiber correspond to different incident pulse energies between 1 and 12 nJ. Measured coupling efficiency was about 24%, which corresponded to a range of energies from 0.24 to 3.8 nJ coupled into the nonlinear fiber. Measured spectra are shown in Figure 8a, together with spectrum of the pump pulse. For low energy pulses up to 4 nJ supercontinuum was registered only with near infrared spectrometer (Ocean Optics NIR512), since signal above 1700 nm was too low with respect to sensitivity of BWTek PbS spectrometer. Supercontinuum generated with input pulses above 7.5 nJ were well above noise level of the spectrometer and were successfully registered. Supercontinuum bandwidth for the highest incident pulse energy spanned from 700 to 3000 nm, which corresponds to two octaves and exceeded expectations set by numerical modeling presented in the previous section. We suppose that supercontinuum bandwidth is presently limited mainly by onset of fiber attenuation in the OH^−^ related absorption peak near 3 µm (Figure 1). Figure 8b shows experimentally and numerically obtained spectra for pump pulse energy of 3.8 nJ (numerical spectrum averaged over 100 shots).

The agreement between simulation and experimental results is reasonable, with few discrepancies, most notably the simulation does not reconstruct full bandwidth at longer wavelengths recorded experimentally, and structure of individual small peaks is different. The differences could originate from a number of reasons. Firstly, dispersion profile in mid-IR range of the real fiber can be different than predicted one, since material dispersion of glass at longer wavelengths is uncertain. Also, we calculated dispersion and effective mode area based on SEM images of drawn fibers, which introduces some inaccuracies (*i.e.*, stemming from thresholding of the SEM image).

The experimental result obtained in this study shows a potential of developed for this fiber for supercontinuum generation. Previously, an octave spanning bandwidth generated pulses in our previous experiment under fiber laser pumping with 400 fs was limited by available peak power of the pump source [15]. Fiber laser with about 2.5 nJ of in-coupled energy of 850 fs pulses was used in [15], and with 2.4 nJ with 400 fs pulses in [16]. Results presented in this work were obtained under pumping with 3.8 nJ of 150 fs pules, which is a factor of roughly 4 to 8 higher peak power with respect to pumping conditions reported previously [15].

It is to be noted, that in this work, measured spectrum is limited on the IR side by the sensitivity of spectrometer used in the experiment. However, a decrease of intensity above 2.9 µm was observed, which was related to increased attenuation caused by OH^−^ absorption. Although transmission of PBG-08 glass is very high with respect to other heavy metal oxide glasses (Figure 1), it is still too low to generate supercontinuum beyond the 2.9 µm.

Spectral flatness of roughly 12 dB is observed in most of the recorded spectrum 900–3000 nm, which is a significant improvement in bandwidth against reported with the previous PCF [14]. Flatness of 5 dB is observed in the spectral range 950–2500 nm, which is similar as previously reported, however input energy of pump pulses was reduced four-fold and at present it outperforms or is at least on par with the other, recently reported supercontinuum results in this part of spectral range [2,3,4].

## 4. Conclusions

A two-octave spanning supercontinuum with a bandwidth of 700–3000 nm, was demonstrated in a lead-bismuth-gallate oxide glass, single-mode, regular lattice photonic crystal fiber. To our knowledge this is the broadest bandwidth reported so far in this type of glasses. The fiber compromise good mechanical properties, time stability and large spectrum generation that covers spectral range to 3 µm important for identification some of the organic compounds. Careful control of fiber modal and dispersion properties with various relative hole sizes in the photonic structure, enabled reduction of input pulse energy four-fold against previously reported result in a fiber made of similar glass. Demonstrated fiber enabled generation of a spectrum, which was flat in a 5 dB dynamic range in an over an octave bandwidth of 950–2500 nm, which predisposes it for practical application in spectroscopy. Supercontinuum bandwidth is presently limited by onset of fiber attenuation in the OH^−^ related absorption area, and possibly a second ZDW, which could be located at a shorter wavelength, than numerically predicted. Technological enhancement of glass purification and control of various relative hole sizes for better long-wavelength dispersion accuracy should allow to extend supercontinuum bandwidth in this fiber design further into mid-infrared.
